# *In Vitro* Exposure of Porcine Ovarian Follicular Cells to PCB 153 Alters Steroid Secretion But Not Their Viability—Preliminary Study

**DOI:** 10.1100/tsw.2002.90

**Published:** 2002-01-29

**Authors:** Ewa L. Gregoraszczuk, Anna K. Wjtówicz

**Affiliations:** Laboratory of Physiology and Toxicology of Reproduction, Department of Physiology, Institute of Zoology, Jagiellonian University, Ingardena 6, 30-060 Kraków, Poland

**Keywords:** polychlorinated biphenyl 153 (PCB 153), early follicular development, steroid secretion cell viability

## Abstract

In our previous paper[1], we demonstrated that porcine follicles collected during the early stage of development are the most sensitive to the toxic action of polychlorinated biphenyl 153 (PCB 153). Follicles of this type were collected to test the effect of PCB 153 on cell steroidogenesis and viability. Cocultures of granulosa and theca cells were grown in M199 medium at 37ºC. Control cultures were maintained in that medium alone, while experimental ones were supplemented with PCB 153 at doses of 5, 10, 50, and 100 ng/ml. After 48, 96, and 144 h, media were collected for steroid analysis and cell viability was measured using an LDH (lactate dehydrogenase activity) cytotoxicity test. A 2-day exposure of follicular cells to all the investigated doses of PCB 153 caused a statistically significant decrease in progesterone (P4) secretion, while in doses of 50 and 100 ng/ml there was also a decrease in testosterone (T) secretion. No effect on estradiol (E2) secretion was observed. The observed decrease in P4 and T secretion, and lack of any statistically significant effect on E2 secretion by cells from small follicles exposed for 48 h to PCB, suggests that PCB 153 acts before P4 formation. Longer exposures caused an increase in P4 secretion, with a concomitant drastic decrease in T secretion and a tendency to decrease the E2 secretion, suggesting inhibition of P450 17*α* hydroxysteroid dehydrogenase, an enzyme that converts P4 to T. The observed PCB 153–induced increase in P4 secretion by cells collected from small antral follicles, with a concomitant decrease in E2 secretion, accounts for the induction of luteinization and, in this case, inhibition of aromatization process in the follicles. However, in all doses tested and at all times of exposure, PCB 153 had no effect on cell viability. These findings suggest different time of exposure–dependent action of PCB 153 on particular steps of steroidogenesis but not action on cell viability. These results should be considered preliminary, pending confirmation by other studies.

### INTRODUCTION

PCBs are persistent environmental pollutants, which exert a variety of toxic effects in animals, including disturbance of sexual development and reproductive function. The overall biological and toxic actions of PCBs, as deduced from animal studies, are rather complex and are not confined to one organ system. It is not always possible to directly and quantitatively predict the occurrence of substance-induced effects from animal data. *In vitro* systems are uniquely suited to investigate specific cellular and molecular mechanisms in the ovary, and thus improve risk assessment. Numerous *in vitro* model systems have been described in the literature. Most of the authors used luteinized granulosa cells collected from superovulated rats or women undergoing *in vitro* fertilization, i.e., not from normal physiological conditions. Enan et al.[2] showed a decrease in P4 production by luteinized granulosa cells after a 24-h exposure to 10 nm of 2, 3, 7, 8-tetrachlorodibenzo-p-dioxin (TCDD). In another study, TCDD was shown to reduce E2 production by luteinized granulosa cells without any effect on P4 production[3].

However, information concerning PCB 153 action on follicular cell steroidogenesis is scarce. To study the direct effect of PCBs on ovarian steroidogenesis in our laboratory, we collected follicular cells from the porcine ovary excised from animals with natural estrous cycles. In our previous paper, we demonstrated that the exposure of porcine follicular cells to PCB 153 influenced steroidogenesis, and that the action of that congener depended on the follicular stage and time of exposure[4,5]. It has been shown that the most sensitive are follicles collected during the early stage of their development[1]. One possible mechanism by which PCBs may interfere with endocrine function is their ability to mimic natural hormones or to act as typical toxic agents influencing cell proliferation and viability. Therefore, in the present study, the objective was to determine whether the observed *in vitro* effect of PCB 153 on steroid secretion was due to its action on steroidogenesis or on cell viability.

### MATERIAL AND METHODS

#### Tissue Preparation and Cultures

Pig ovaries collected during the early follicular phase of the estrus cycle were obtained from a slaughterhouse. The separation of Gc from the theca layer was performed according to the technique described by Stoklosowa et al.[6]. Granulosa and theca cells were inoculated at a concentration of $6.8 \times 10^6$ and $2.1 \times 10^6$ cells/ml, respectively, and thus at a concentration comparable to that observed *in vivo* (Gc: Tc = 3:1). Cells were cultured in Parker Medium (M199) supplemented with 10% calf serum with or without PCB153. The cultures were maintained at 37°C in a humidified atmosphere of 5% $CO_2$. Parker Medium M199 and calf serum were purchased from the Laboratory of Sera and Vaccines, Lublin, Poland. Stock solution of PCB 153 (2,2', 4,4', 5,5'-CB; 25 μg/ml) was prepared by dissolution of the pure powder in ethanol (Prochem GmbH, Wesel, Germany; purity 0.997).

To test PCB 153 action on steroids, secretion cells were plated into 24 well plates for 48-, 96-, and 144-h incubation, with M199 supplemented with 5% of calf serum as a control medium or incubated with different doses of PCB 153 (5, 10, 50, and 100 ng/ml). After 48, 96, and 144 h, all cultures were terminated and the media were frozen until further steroids analysis.

#### *Steroids Analysis*

P4, T, and E2 levels were determined by radioimmunoassay using Spectra kits (Orion, Diagnica, Finland) supplied by Polatom (Świerk, Poland). The lowest sensitivity level of the P4 assay was 94 pg/ml, and the coefficients of variation between and within assays were 5.8 and 2.9%, respectively. The mean recoveries were 95.1–103.7%. Cross-reactivity with pregnenolone was 2.9%, whereas other tested steroids such as 5β-dihydroprogesterone, 20β-hydroxyprogesterone, corticosterone, T, and estrone showed less than 1% cross-reactivity. The limit of the T assay's sensitivity was 5 pg/ml, and the coefficients of variation within and between assays were 5.4 and 5.3%, respectively. The mean recoveries were 84.2–121.7%. Cross-reactivity with 5α-dihydrotestosterone was 4.5%. All other tested steroids (methyltestosterone, androstendione, P4, and 17β-estradiol) showed less than 0.5% cross-reactivity. The detection limit of E2 assay was 5 pg, and the coefficients of variation between and within assays were 10.28 and 2.9%, respectively. The mean recoveries were 85.6–108.9%. The cross-reactivity with ethinyl E2 was 1.4%. All other tested steroids (estrone, estriol, P4, T, and corticosterone) showed less than 1% cross-reactivity.

### Cell Viability

#### Lactate Dehydrogenase Cytotoxic Assay

Lactate dehydrogenase activity (LDH) was determined by the calorimetric method according to the technique of Cabaud and Wroblewski[7]. The reduction of pyruvate to lactate is proportional to the amount of LDH, and the pyruvic acid reacts with 2,4-dinitrophenylhydrazine to form a hydrazone. The absorbency at 490–500 nm was measured. The amount of pyruvic acid remaining after incubation is inversely proportional to the amount of LDH in the samples of culture medium.

### Statistical Analysis

All data points are expressed as means ± SEM from at least three different experiments (n = 3), each in triplicate. The significance of differences between the concentrations of P4, E2, and T in the control and experimental cultures was compared by analysis of variance using Duncan's multiple range test.

## RESULTS

Two days exposure of follicular cells to all investigated doses of PCB 153 caused a statistically significant decrease in P4 secretion (56, 46.9, 46.8, and 47.9% of the control culture, respectively, after 5, 10, 50, and 100 ng/ml of PCB 153; $p < 0.05$). In doses of 50 ng and 100 ng/ml, it also caused decreased T secretion ($p < 0.05$). No effect on E2 secretion was observed (Fig. 1a–c).

Longer exposures to PCB caused an increase in P4 secretion (129, 135, and 123% of the control cultures after 96-h exposure to 10 ng, 50 ng, and 100 ng/ml of PCB; $p < 0.05$; and 260%, 260% of control after 144-h exposure to 50 ng and 100 ng/ml of PCB; $p < 0.001$) (Fig. 1a). A concomitant, drastic decrease in T secretion after 96-h exposure (58%, 50% of control after 5 ng, 10 ng; $p < 0.01$; and 9%, 9% of the control after 50 ng and 100 ng/ml of PCB; $p < 0.001$) and 144-h exposure (30% of control after 10 ng PCB; $p < 0.01$; and 5%, 5% of control after 50 ng and 100 ng/ml PCB; $p < 0.001$) was noted (Fig. 1b). A 144-h exposure to PCB caused a decrease of E2 secretion (68 and 72% of control after 10 and 50 ng PCB; $p < 0.05$; and 49% of control after 100 ng PCB; $p < 0.01$) (Fig. 1c).

In all doses used in this experiment, and during all time exposures, *in vitro* PCB 153 had no effect on follicular cell viability measured by lactate dehydrogenase cytotoxic assay (Fig.1a–c).

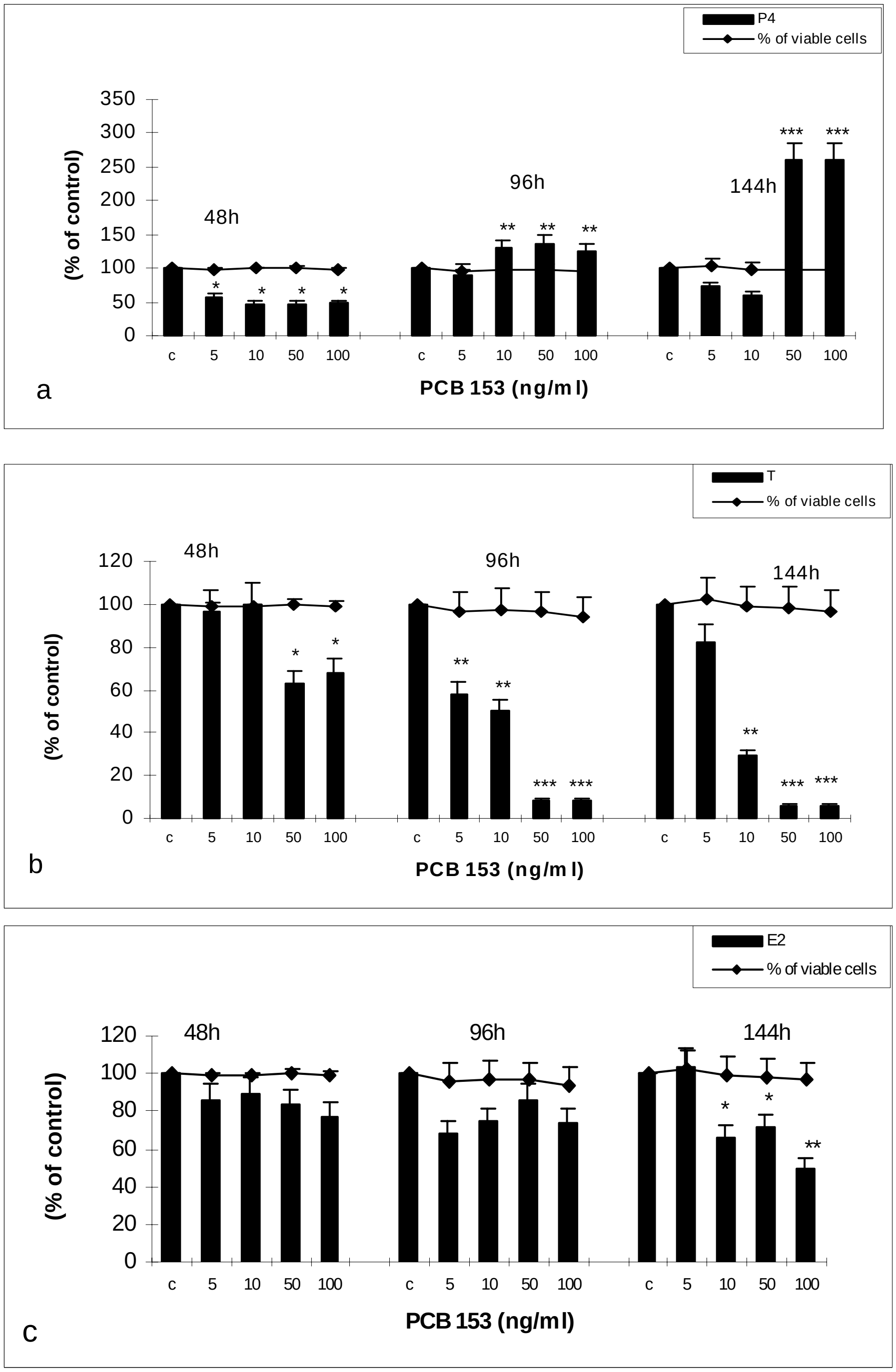

**FIGURE 1.** Dose-dependent effect of PCB 153 on follicular cell viability and steroids secretion: (a) P4, (b) T, and (c) E2. All values are the mean +/- SE and expressed as percent secretion with the control as 100*. * = <0.05; ** = $p < 0.01$; *** = $p < 0.001$.

### DISCUSSION

The present data clearly show that PCB 153 has a direct effect on follicular steroidogenesis. The observed decrease in P4 and T secretion, with no statistically significant effect on E2 secretion by small follicles exposed for 2 days to PCB, suggests action on cholesterol mobilization as a precursor for steroids biosynthesis and then action on cytochrome P450scc before P4 formation. This mechanism of action was suggested for dioxin acting as an endocrine disrupter in follicular cells[8]. Richards[9] showed an atretogenic action of T on preantral follicles.

The observed PCB 153–induced increase in P4 secretion by cells exposed for 144 h with a concomitant decrease in T and E2 secretion accounts for induction of luteinization and, in this case, inhibition of aromatization process.

Previously it has been shown that high levels of P4 might have an inhibitory effect on aromatase activity[10,11]. On the other hand, 96- and 144-h exposures of follicular cells to PCB caused a drastic decrease in T secretion and a tendency to decrease E2 secretion, suggesting action on P450c17 $\alpha$, an enzyme that converts P4 to T and, as a consequence, decreasing the substrate for E2 production by follicular cells. Taking into consideration that during the early stage of follicular differentiation androgen acts as an enhancer of FSH-stimulated follicular differentiation, the failure in this process observed under the influence of PCB 153 might result in inappropriate androgenic stimulation, and consequently follicular atresia. PCBs often referred as endocrine disrupters are thought to mimic endogenous estrogens by entering the cells, binding to the receptor and activating transcription. They may also antagonize normal androgen action[12].

Concentration-dependent decreases in P4 secretion after short-term exposure to PCB 153 and concentration-dependent increases in P4 secretion after long-term exposure were observed in cultured luteal cells collected from pigs in the middle-luteal phase[13].

The second interesting finding of the presented study was the lack of a relationship between PCB exposure and cell viability. PCB 153 in all doses used, and all times of exposure, had no effect on cell viability (Fig. 1a–c). This finding is not in accordance with the results of Rogers[14], who showed that compared to the total concentrations found in untreated controls, CHO-K1 cells (a sensitive indicator of the cytotoxicity of PCBs) treated with Aroclor 1016 contained less neutral lipid and more phospholipid. They suggested that changes in membrane neutral lipid and phospholipid components observed at marginally cytotoxic levels of Aroclor provided evidence that PCBs may indeed affect membrane integrity and associated metabolic functions.

In a recent study conducted in our laboratory, PCB 153 in doses of 5 and 10 ng/ml had no effect on cells viability during all investigated periods of exposure. However, a decrease in cell viability to 52 and 48% of the control after 72-h exposure to 50 and 100 ng/ml was observed. We suggest that, during longer exposure, this congener causes an increase in cell membrane permeability resulting in increased steroid outflow into the medium[9].

It is, however, difficult to compare these other data with ours because different models were used in these studies. Rogers[14] used the CHO-K1 cell line and Augustowska[13] used luteal cell cultures, while in the present data, we primarily used a coculture of granulosa and theca cells. This suggests that toxicity of PCB 153 is dependent upon cell type.

For the ortho-substituted congeners, the link to specific toxic endpoints is not well established, and it is likely that multiple mechanism are involved[15]. Toxicity could result from over- or underexpression of normal hormonal responses that may be mediated by both receptor and nonreceptor protein and associated DNA interactions[16].

Although ortho-substitution in PCBs can significantly lower the dioxin-like toxicity, it can potentially have the opposite effect on any estrogen-receptor-mediated toxicity. Recently, Bonefeld-Jorgensen et al.[17] presented evidence that PCB 153 has pleiotropic effects on the estrogen and androgen receptor. They indicated that PCB 153 can compete with the binding of the natural ligand to two nuclear receptors, and thus possesses the ability to interfere with sexual hormone-regulated processes.

Dose-dependent changes in steroids secretion under the influence of PCB 153, with no effect on cell viability, suggest that in our cell culture systems PCB 153 acts as an endocrine disrupter and not as a toxin per se. Other mechanisms of potential interference with endocrine functions exist, including effects on enzymes involved in steroid synthesis and metabolism. To confirm the actions of these congeners as typical endocrine disrupters, further studies are necessary to show the actions on particular steps of steroidogenesis and estrogen-mediated activity of PCB 153 in our cells culture condition. *De novo* synthesis of 17β-estradiol starts with the conversion of cholesterol to pregnenolone by CYP11A (cholesterol side-chain cleavage). In the subsequent steps, 3β-SHD, CYP17 (17β-hydroxylase and 17,20 lyase activity), 17β-HSD, and CYP19 (aromatase) are involved[18]. CYP 19 is of particular interest, as it is the rate-limiting catalyst in the formation of estrogen. In addition, enzymes that metabolize steroid hormones regulate steroid homeostasis[19]. The isoforms of CYP enzymes involved in estrogen hydroxylation are species- and tissue-dependent. Cytochrome P4501A1 (CYP1A1) is involved in the metabolism of steroid hormones and polycystic aromatic hydrocarbons in humans[20]. However, induction of CYP1A1 in mammalian cell cultures is widely used as a functional parameter for AhR activation, providing an estimate for “dioxin-like”-inducing equivalents in extracts from environmental samples[21].

In conclusion, the presented data suggest that disruption of follicular steroidogenesis is not due to its action on cell viability, but to the disruption of particular steps of steroidogenesis. Moreover, it suggests that this action depends on the time of exposure. These results should be considered preliminary, pending confirmation by other studies.

### ACKNOWLEDGEMENTS

We are grateful to Dr. M. Mika from the Department of Physiology, Academy of Agriculture, Krakow, Poland, for steroid analysis. This work is supported by the grant KBN 5 PO6D/010/18.

### BIOSKETCH

**Ewa L. Gregoraszczuk** is Professor of Biology and Head of Laboratory of Physiology and Toxicology of Reproduction at the Institute of Zoology, Jagiellonian University, Kraków, Poland. A biologist and endocrinologist, Prof. Gregoraszczuk's research interests lie in growth hormone and leptin action on ovarian steroidogenesis, dioxin and polychlorinated biphenyls as an endocrine diruptors, valproate-antiepileptic drug. She is currently working on the field of physiology and toxicology of reproduction. In the field of physiology of reproduction she is interested in growth hormone and IGF-I synergistic action on ovarian follicular cells steroidogenesis. Her lab is working on the effect of leptin as a factor speed up sexual maturity. In the field of toxicology of reproduction she investigated accumulation of dioxin and polychlorinated biphenyls in the ovary and placenta and as a consequence action on ovarian and placental steroidogenesis. In cooperation with Norwegian partners she is investigating the effect of valproate on ovarian dysfunction.
